# Epidemiological study of prostate cancer (EPICAP): a population-based case–control study in France

**DOI:** 10.1186/1471-2407-14-106

**Published:** 2014-02-19

**Authors:** Florence Menegaux, Antoinette Anger, Hasina Randrianasolo, Claire Mulot, Pierre Laurent-Puig, François Iborra, Jean-Pierre Bringer, Benoit Leizour, Rodolphe Thuret, Pierre-Jean Lamy, Xavier Rébillard, Brigitte Trétarre

**Affiliations:** 1 INSERM U1018, Center for Research in Epidemiology and Population Health (CESP), U1018, Environmental Epidemiology of Cancer Team, 16av. Paul Vaillant Couturier, 94807 Villejuif Cédex, France; 2 Univ Paris-Sud, UMRS 1018, 16av. Paul Vaillant Couturier, 94807 Villejuif Cédex, France; 3 INSERM U1147, Paris, France; 4 Univ Paris Sorbonne Cité UMRS 775, Paris, France; 5 Cabinet Urologie du Polygone, Montpellier, France; 6 Service d’Urologie, Hôpital Lapeyronie, Centre Hospitalo-Universitaire de Montpellier, Montpellier, France; 7 Polyclinique Saint Privat, Béziers, France; 8 Service Urologie, Clinique Beau Soleil, Montpellier, France; 9 Laboratoire de Biologie Spécialisée et Oncongénétique, Institut du Cancer de Montpellier, Montpellier, France; 10 Registre des Tumeurs de l’Hérault, Montpellier, France

## Abstract

**Background:**

Prostate cancer is the most common cancer in male in most Western countries, including France. Despite a significant morbidity and mortality to a lesser extent, the etiology of prostate cancer remains largely unknown. Indeed, the only well-established risk factors to date are age, ethnicity and a family history of prostate cancer. We present, here, the rationale and design of the EPIdemiological study of Prostate CAncer (EPICAP), a population-based case–control study specifically designed to investigate the role of environmental and genetic factors in prostate cancer. The EPICAP study will particularly focused on the role of circadian disruption, chronic inflammation, hormonal and metabolic factors in the occurrence of prostate cancer.

**Methods/Design:**

EPICAP is a population-based case–control study conducted in the *département* of Hérault in France. Eligible cases are all cases of prostate cancers newly diagnosed in 2012-2013 in men less than 75 years old and residing in the *département* of Hérault at the time of diagnosis. Controls are men of the same age as the cases and living in the *département* of Hérault, recruited in the general population.

The sample will include a total of 1000 incident cases of prostate cancer and 1000 population-based controls over a 3-year period (2012-2014).

The cases and controls are face-to-face interviewed using a standardized computed assisted questionnaire. The questions focus primarily on usual socio-demographic characteristics, personal and family medical history, lifestyle, leisure activities, residential and occupational history. Anthropometric measures and biological samples are also collected for cases and controls.

**Discussion:**

The EPICAP study aims to answer key questions in prostate cancer etiology: (1) role of circadian disruption through the study of working hours, chronotype and duration/quality of sleep, (2) role of chronic inflammation and anti-inflammatory drugs, (3) role of hormonal and metabolic factors through a detailed questionnaire, (4) role of individual genetic susceptibility of genes involved in biological pathways of interest. The EPICAP study will also allow us to study prognostic factors and tumor aggressiveness.

Taken together, the EPICAP study will provide a comprehensive framework to go further in the understanding of prostate cancer occurrence and its prognosis.

## Background

In most Western countries, prostate cancer (PCa) is by far the most commonly diagnosed cancer in men, which represents, in France, an estimated 71,220 new cases and 8,685 deaths in 2011 [1]. However, this estimate was revised down by the French network of cancer registries FRANCIM, with an estimate closer to 60 000 new cases per year in France for 2012. A rise in incidence has been observed in the majority of Western countries, including France (+8.5% over the period 2000-2005) partially due to the massive increase in screening by PSA assay (Prostate-Specific Antigen) since the beginning of the 1990s. Interestingly, this rise in incidence is also beginning to be observed in countries where the incidence was very low, especially in Asia [2].

Despite a relatively high morbidity and mortality (3rd leading cause of cancer death in France), only age, ethnicity and a family history of prostate cancer are well-established risk factors of prostate cancer, and except those factors, the etiology of prostate cancer remains largely unknown [3,4]. The incidence of prostate cancer varies considerably with geographical location and ethnic origin with highest incidence rates observed in Afro-American and French West Indian populations (> 170 new cases per 100,000) and lowest incidence rates observed in Asians (< 20 new cases per 100,000). The majority of Westernised countries, including France, have incidence rates that vary between 60 and 100 new cases per 100,000.

Interestingly, migrant studies have shown that Asian men living in the USA have much higher PCa rates than their counterparts living in native lands, and the incidence of PCa in previously low-risk Asian countries has increased in parallel to affluence [5]. This suggested the importance of the environment and lifestyle factors in prostate cancer etiology and pathogenesis [6,7].

Current research on prostate cancer etiology include the role of the environment, chronic inflammation, hormones and metabolism, diet and genetic factors with a growing interest in the complex interrelationships that may exist between these factors. This is to answer some of these research questions that we implemented the EPICAP study, a population-based case–control study, whose main objective is to investigate the role of environmental and genetic factors in the occurrence of prostate cancer. A secondary objective will investigate some prognostic factors and tumor aggressiveness.

The EPICAP study will particularly focused on the role of circadian disruption, chronic inflammation, hormonal and metabolic factors and genetic factors in the occurrence of prostate cancer.

### Circadian disruption and prostate cancer risk

The hypothesis that night work or rotating shift work may play a role in the occurrence of cancer was raised twenty years ago trying to explain, at least in part, the increase in breast cancer incidence in industrialized countries.

Following the publication of several epidemiological studies, the International Agency for Research on Cancer (IARC) has classified in 2010 the “shift leading to a disruption of circadian rhythm” as probably carcinogenic to humans (Group 2A) on the basis of sufficient evidence in animals and limited evidence in humans [8].

Disruption of circadian rhythm may increase the risk of cancer, either by direct disruption of the circadian clock genes function that control cell proliferation or due to the disruption of the clock-controlled settings such as melatonin levels or sleep disorders [9-11]. This hypothesis was mainly investigated in hormone-related cancers in women (breast, ovary, endometrium), and studies regarding the potential effects of circadian disruption on prostate cancer risk are scarce [12]. Only five studies have examined the association between night work and prostate cancer with discordant results [13-16]. A high incidence of prostate cancer has also been observed among pilots in a meta-analysis [17]. Finally, one study reported a decreased risk for those sleeping at least 9 hours per night [18]. This result is consistent with the fact that nocturnal melatonin secretion is more important when sleep duration is long and that melatonin has oncostatic effects of its own.

### Chronic inflammation, non steroidal anti-inflammatory drugs and prostate cancer risk

Chronic inflammation has been associated with the development of several cancers via a specific infectious or environmental agent [19-21]. The presence of inflammatory infiltrates located near areas of proliferative inflammatory atrophy (PAI) and prostatic intraepithelial neoplasia (PIN), considered as precancerous prostate lesions, also helped to strengthen the hypothesis of a possible link between chronic inflammation and prostate cancer [22-24]. Several studies have observed an association with a personal history of prostatitis or sexually transmitted infection [25,26]. Sexual activity, as a marker of androgenic activity due to high sexual activity or a marker of sexually transmitted infection caused by a number of partners, has also been associated with prostate cancer [27]. In addition, some studies have reported elevated plasma levels of pro-inflammatory cytokines or immune response inhibiting cytokines in men with prostate cancer [28-30]. Finally, some polymorphisms of genes encoding cytokines have also been associated with prostate cancer [31-33].

All these elements led to the hypothesis of a possible reduction in the risk of cancer through the use of non steroidal anti-inflammatory drugs (NSAIDs) [34-36]. Various mechanisms have been proposed: induction of apoptosis, inhibition of angiogenesis, and inhibition of growth by direct inhibition of cyclooxygenase-2 (COX-2). As in mammary tumor cells, overexpression of COX-2 was also observed in prostate tumor cells [37,38], and higher levels of prostaglandins have been detected in malignant prostate tissue compared to benign prostate tissue [39]. Several epidemiological studies report results in favor of a slightly decreased risk of prostate cancer with aspirin consumption but very few studies have looked at other NSAIDs, including selective inhibitors of COX-2 [40,41].

### Hormones, metabolism and prostate cancer risk

Androgens (testosterone, dihydrotestosterone and their derivatives) play a central role in the development and growth of the prostate, however, the precise role of androgens in the development of prostate cancer is far from clear [42,43]. Indeed, epidemiological studies have shown conflicting results regarding the role of circulating androgens or other sex hormones in the development of prostate cancer [44-47]. Interestingly, the role of several markers of hormonal impregnation such as male premature pattern baldness, history of acne in childhood or teenage years and hypofertility/infertility have also been explored in prostate cancer pathogenesis with contradictory results [48-51].

Clinical, molecular and epidemiological studies have provided emerging evidence of a possible role of obesity, involving complex biological pathways such as the hypothalamic-pituitary-adrenal (HPA) axis, growth factors, the pancreas and the sympathetic nervous system, peptide hormones such as leptin and insulin, and more recently the metabolic syndrome in prostate cancer pathogenesis [6,52-55]. Although insulin resistance and obesity are considered at the core of the pathophysiology of metabolic syndrome, a number of other factors, such as chronic stress and dysregulation of the hypothalamic-pituitary-adrenal (HPA) axis, circadian clock system, proinflammatory state and cellular oxidative stress can also be involved in its pathogenesis [56]. Therefore, several mechanisms could explain the association of obesity and metabolic syndrome with prostate cancer pathogenesis, including sex steroid hormone, insulin and insulin-like growth factors and inflammation pathways [6,57-59].

Considering the possible link between obesity, metabolic syndrome and prostate cancer, it has been suggested that prevention through lifestyle intervention and treatment of those conditions may reduced the risk of prostate cancer. Therefore, the possibility of influencing prostate cancer risk through therapies that alter cholesterol metabolism such as statins, cholesterol-lowering drugs, has also been investigated with controversial results [60-62].

### Genetic factors and prostate cancer risk

Familial aggregation is now well established with an increased risk of prostate cancer in patients with a family history of prostate cancer in first degree relatives [63]. This risk is even more important if the related is a brother and if the number of affected relatives is large.

Family studies have also shown that some prostate cancers can be inherited as an autosomal dominant model and it has been estimated that prostate cancer family, due to a rare gene with high penetrance, accounted for approximately 10% of all prostate cancer [64]. Seven loci have been described, identified and located on several genes, especially RNASEL/HPC1, ELAC2/HPC2 and MSR-1 [64-67].

Some polymorphisms of genes involved in the metabolism of steroid hormones (HSD17B, SRD5A2, CYP17, CYP19, COMT), in the metabolism of xenobiotics (CYP1A1, CYP1A2, CYP1B1, GSTP1) and in circadian rhythms (PER1, PER2, CRY1, CRY2, CSNK1E, ARNTL, CLOCK) have also been associated with the occurrence of prostate cancer [68-71]. In addition, gene polymorphisms of circadian rhythms have also been associated with high concentrations of sex steroid hormones, suggesting a biological support for the role of genes in the circadian rhythm of hormone-dependent cancers [72].

### Prognostic factors and tumor aggressiveness

PSA is the leader biomarker for early detection, diagnosis, prognosis and follow-up in prostate cancer. However, at the time of diagnosis, if high levels of PSA are related with more aggressive disease, moderate elevation of PSA cannot distinguish patients with a risk of relapse to patients with low risk of recurrence and mortality, those patients deserving active surveillance and not possibly harmfully treatment. A well-developed prospective biobank is mandatory for high-quality research of new tumor markers. Moreover, the recent development of Liquid Chromatography–Mass Spectrometry (LC-MS) technology and Reverse Phase Protein Microarray (RPMA), a high-density quantitative, calibrated, multiplexed array for protein analysis allows us to develop specific proteomic signatures for PCa prognosis or related to the aggressiveness of the disease.

## Method/Design

We have implemented a population-based case–control study in the département of Hérault in France. This is the most suitable type of study for the simultaneous analysis of several risk factors. A population-based study achievement through an exhaustive recruitment of the cases in a well defined geographic area and the recruitment of the controls in general population offers the advantage of preventing selection problems as much as possible.

The Département of Hérault was chosen owing to the existence of a general cancer registry created 26 years ago (http://www.registre-tumeurs-herault.fr) and of particular interest for prostate cancers through collaborative projects with the Regional Association for Clinical Research and Consensus in Onco -Urology in Languedoc-Roussillon– ARCOU (a federation of nearly all urologists, oncologists, radiotherapists, pathologists, researchers and epidemiologists of Languedoc Roussillon, some of whom are from the register), because of its agricultural and urban character, and because of its large size (over one million inhabitants), which allows the validation of large numbers of cases (~ 900 validated cases per year) and the collection of data over a relatively short time period for an epidemiological study. The Hérault department is involved in the European randomized Study of screening for prostate cancer (ERSPC) study [73,74].

### Cases selection

Eligible cases are all patients newly diagnosed with prostate cancer in 2012-2013, less than 75 years old and resident in the department of Hérault at diagnosis. The case identification is performed by clinical research nurses recruited and trained specifically for the study in all participating centers: 3 public hospitals and 3 private urology clinics. Each clinical research nurses has been assigned one or more specific health care facilities. Each clinical research nurses has been assigned one or more specific health care facilities. The registry allows us to validate the cases that have been included in the study. The inclusion of patients in the study was made only after collecting their consent. Only histologically confirmed cancer cases were included in the study.

### Controls selection

Controls were selected among general population men free of cancer and resident in the study area (département of Hérault) at the time of the cases’ diagnoses. For including controls, quotas by age were established as a preliminary to yield the control group similar to the case group in terms of age in order to achieve frequency-matching (5 year age group). Quotas by socio-economic status (SES) were also set a priori to control for potential selection bias arising from differential participation rates across SES categories. These quotas by SES were calculated from the census data available in each study area, in order to obtain a distribution by SES among controls identical to the SES distribution among general population men, conditionally to age. The recruitment of controls was conducted as follows: phone numbers of private homes were selected at random via a survey institute (selected by public procurement) from the telephone directory of the study area where unlisted numbers had first been re-created. A phone number was dialed up to 20 times at different times of the day and different days of the week until contact could be established with the residents. When a man was living in the residence reached by phone, he was invited to participate to the study, as long as the predefined quota corresponding to his age group and socioeconomic status (SES) was not completed. When the quota was exceeded, the man was excluded. The control list of men who accepted to participate to the study is provided to the research team on a monthly basis and the controls are contacted within 2-3 days by clinical research nurses.

Overall, the EPICAP study will include 1000 prostate cancer incident cases and 1000 population-based controls over a three years period (2012-2014).

### Organization

A steering committee, including the coordination team of the study (principal investigator and study monitor), the director of the Hérault Cancer Registry (Brigitte Trétarre) and a referent for urologists (Xavier Rébillard) was set up. This committee meets every 3-4 months with the three research clinical nurses in order to maintain nurses’ motivation and training and to follow the case and control recruitment as good as possible with the different partners involved in the study.

The study monitor, Hasina Randrianasolo, is responsible for the coordination of the 3 clinical research nurses and for recruitment follow-up, for checking eligibility and inclusion criteria as well as the receipt of consent, for the follow-up of interviews of cases and controls, for the follow-up of samples together with the Biological Resources Center Epigenetec in Paris and the specialized biology laboratory of the cancer institute of Montpellier, for the constant updating of the database for the management of the investigation and for drawing up reports on the state of advancement of the study.

The 3 clinical research nurses have been specifically trained for this study, particularly in the use of the CAPI system and with respect to the occupational questionnaires. They are in charge of identifying cases in real time in collaboration with the physicians and the registry network, of including cases into the study after obtaining their consent, of conducting face-to-face interviews with the cases and controls as well as taking biological samples from the cases and controls, and of sending the samples to the Biological Resource Centre in paris and the specialized biology laboratory in Montpellier. Each day, the clinical research nurses are in charge of sending the computerized questionnaire to the survey institute using an internal network. They are also in charge of sending the occupational questionnaires to the research team weekly.

The survey institute (selected by public procurement), under the supervision of the research team, has been in charge of the computerization and CAPI formatting of the study questionnaire, of setting up the database for the selection of controls by drawing lots through the geographically random generation of telephone numbers. It is also in charge of selecting the control group according to the study protocol by complying with the recruitment of monthly quotas and ensuring that consent is obtained, of providing monthly reports on the quotas and the recruitment of controls.

The cancer register of the Department of Hérault plays a central role in the organisation of the study. Indeed, the study relies on the network of physicians in the *département*, set up by the register, who are federated around the Association for Clinical Research in Onco-Urology (ARCOU).

The Biological Resource Centre – Epigenetec, located in Paris, is in charge of the reception, recording (DATABIOTEC software), management and storage of biological samples (EDTA blood samples or Oragen® saliva kits) from cases and controls.

The specialized biology laboratory in Montpellier is in charge of the reception, recording, management and storage of the dry tubes blood samples from cases and controls. This lab will be responsible for the study of prognostic factors and tumor aggressiveness under the supervision of Dr Pierre-Jean Lamy.

### Data collection

Cases and controls are face-to-face interviewed by an experienced research clinical nurse especially trained for this study and are undergo anthropomorphic measurements. The conditions for data collection are the same for cases and controls. A blood sample or failing that, a saliva sample is proposed but refusal does not constitute a motive for exclusion.

– Questionnaire (Additional file 1)

– The interview with cases and controls are carried out using a system-standardised questionnaire (CAPI - Computer Assisted Personal Interview), a face-to-face method for collecting data on a micro-computer in the home of the interviewee. The interview lasts 2 to 4 hours. The interview allows the gathering of information concerning the usual socio-demographic characteristics, a full professional and residence history, lifestyle and leisure activities, as well as a personal and family medical history. Ethnic origin is also carefully recorded.

– Data concerning *environmental factors* include a full history of the various places of residence, lifestyle (tobacco and alcohol consumption, Morningness-Eveningness Questionnaire, duration and quality of sleep) as well as leisure activities such as gardening, painting and physical activity.

– Data concerning *occupational factors* include a detailed occupational questionnaire retracing the full employment history. For each type of employment (of more than 6 months), individuals will be asked for the starting and termination dates, a description of the job and tasks involved, and the name and address of the company. Certain professions (farmers, professional users of pesticides, food or rubber industry workers, medical workers, night watchers…) and certain working hours (night work, rotating shift work) are more specifically documented using specific occupational questionnaires. The occupational exposure assessment will be performed using the occupational history questionnaire with job-exposure matrix or a case by case evaluation by an industrial hygienist expert.

– The *infectious and inflammatory factors* are recorded by detailed questioning on hospitalizations, surgery, infectious diseases (uro-genital infections, sexually transmitted infections…), inflammatory diseases, medication (non steroidal anti-inflammatory drugs, antibiotics…).

– Data on *hormonal and metabolic factors* are collected via questions on the individual’s personal medical history (diabetes, arterial hypertension, dyslipidemia, metabolic syndrome…), hormonal maturation (puberty, acne, Norwood-Hamilton scale of baldness), medication (anti-diabetic, anti-hypertensive, cholesterol-lowering drugs, testosterone, etc.), fertility (conception problems, fatherhood status…), weight history. A food frequency questionnaire is also completed.

– The family history of cancer in 1st degree and 2nd degree relatives is examined. For each affected relative, the localization and age of occurrence of the cancer is recorded. A full description of 1st and 2nd degree relatives is recorded in order to take into account the family structure in the analyses.

– Anthropomorphic measurements

– The nurses also carried out anthropomorphic measurements (weight, height and abdominal and hip perimeter) for both cases and controls. These measurements complement the data obtained using the questionnaire.

– Biospecimen collection

– Blood sampling is proposed to both cases and controls and is carried out after signing informed consent (two dry tubes of 5 ml and two tubes of 7 ml with EDTA as anticoagulant). In case blood sampling is refused, saliva sampling is proposed instead, using an Oragene® kit. Blood sampling allows the constitution of serum and DNA banks.

– Cases also gave their consent for the use of their tumour samples in case of surgery. This allows the constitution of a tumour bank. Projects which may be implemented from the tumour bank will be done in collaboration with all pathologists of the *département*.

– Clinical data

– The medical data for all prostate cancer cases are collected by the clinical research nurses in medical record of each case in collaboration with the urologists of each medical department and the cancer registry of the *Département* of Hérault.

– Data management

– The computerized questionnaires from cases and controls are monthly returned to the research team by the survey institute in the form of SAS files. The data manager is then responsible for checking the files provided, controlling data quality by setting up logical checks (answer consistency and outlier search).

### Study power

The size of the study will enable detection of minimum odds ratios of 1.3, 1.4 and 1.7 for exposure whose prevalence in controls are 30, 10 and 5 percent, respectively, with a power of 80 percent, a type-I error of 5 percent and a number of 1000 cases and 1000 controls.

Based on a literature review and results of previous studies, prevalence of worthwhile factors is expected to be mostly more than 5 to 10% in general population for specific medical conditions or medication of interest and even being around 35 to 45% for men involved in rotating shift and night work.

### Ethical aspects

Each subject enrolled in the study provides his written informed consent. In order to protect the confidentiality of personnel data and to fulfil legal requirements, the questionnaire included only an identification number, without any nominative information. The same identification number is used for biological samples. The link between the subject’s name and the identification number is kept by the research team.

The EPICAP study was approved by the Institutional Review Board of the French National Institute of Health and Medical Research (IRB-Inserm n° 01-040 - November 2010) and by the French data Protection Authority (CNIL N° 910485 - April 2011).

In France, for epidemiological studies that are not clinical trials, the ethical approval is a global approval for the study based on the list of participating centers that are described in the protocol. We also obtained the approval of all participating centers and approval of all urologists of these centers.

### Pilot study

We conducted a pilot study from June 2011 to January 2012 which allowed us the set up the study in term of: computerization of the study questionnaire, fluency of the questionnaire, testing of the questionnaire, setting up the database for the selection of controls by drawing lots through the geographically random generation of telephone numbers, hiring 3 clinical research nurses, identifying cases in real time in collaboration with the Hérault cancer registry and physicians, subject’s acceptance of the study questionnaire and biological samples. The pilot study included 61 cases and 121 controls with a participation rate of 80% for both cases and controls. All subjects but four (1 case and 3 controls) accepted the biological sample.

## Discussion

EPICAP is large a population-based case–control study designed to assess the role of environmental and genetic factors in prostate cancer. EPICAP is drived in *département* of Hérault by INSERM Unit 1018 with all urologists and pathologists involved in the diagnosis and treatment of prostate cancer cases. This is the most suitable type of study for the simultaneous analysis of several risk factors. A population-based study achievement through an exhaustive recruitment of the cases in a well defined geographic area and the recruitment of the controls in general population offers the advantage of preventing selection problems as much as possible.

Case identification is based on the network of investigators working with the Hérault Cancer Registry and all health care facilities that support prostate cancer patients allowing the exhaustiveness of case recruitment of the study. In order to minimize selection biases, cases were identified in all cancer hospitals, either public or private, that recruited prostate cancer patients in the *département* of Hérault. Controls are selected from the general population in the *département* of Hérault, using quotas defined for age and socioeconomic status (SES). The age distribution of the controls reflects the age distribution of the cases. In order to avoid selection biais, the SES distribution of the control group reflects the SES distribution of the entire *département* of Hérault to yield the control group similar to the general population of men of the same age in terms of SES.

To minimize recall bias that may not completely ruled out in case–control studies, data were collected by the same research clinical nurses for cases and controls using a standardized questionnaire and in the same conditions of interviewing for both cases and controls. Using the CAPI (Computed Assisted Personal Interview) system presents the advantage of not having to spend time on coding.

The main objective of the EPICAP study is to particularly focus on the role of circadian disruption, hormonal, metabolic and inflammatory factors in the occurrence of prostate cancer.

Epidemiological studies that have examined the role of circadian disruption in the development of cancers through night work bring up two major problems: assessment and quantification of exposure in one hand, and taking into account other risk factors in the other hand. Exposure to night work or shiftwork was often determined on the basis of a simple classification of subjects “Ever/Never” or based on specific occupational cohorts. Moreover, the vast majority of the literature on the role of night work in cancer mainly focuses on breast cancer and very few studies has been published on this subject for prostate cancer. The study of the role of night shift work in prostate cancer is of major importance in terms of public health due to the increase in the number of men working at night or with rotating and staggered schedules in modern societies. Indeed, 35 to 45% of European men had staggered working hours, including night work, in 2005 [75]. The study of the role of circadian disruption goes beyond the study of night work. There is also a need to take into account the chronotype of the subject as well as the quantity and quality of sleep.

Given the rising epidemic of obesity and metabolic syndrome worldwide, especially in developing countries, and the potential links among obesity, androgen metabolism, metabolic syndrome and inflammation, it is critical to better understand the complex relations between overall and abdominal obesity and prostate cancer risk and the role of chronic inflammation in prostate cancer pathogenesis. The act of gathering all these factors of interest in a given study is essential in order to simultaneously take into account all these factors.

The identification of polymorphisms that predispose to prostate cancer and their interaction with “epidemiological” factors constitutes a major issue in order to better understand the mechanisms of prostate cancer development.

In conclusion, EPICAP is a large population-based case–control study and the first study of this magnitude in France mainland. Its main objectives are to investigate several suspected risk factors whose interest has been growing worldwide in the last decade. This study was conceived as part of a comprehensive approach defined recently as “integrative epidemiology” by Spitz and collaborators [76,77]. Indeed, the EPICAP study collects clinical, biological, epidemiological and genetic data which will provide a comprehensive framework to go further in the understanding of prostate cancer occurrence and its prognostic.

## Competing interests

The authors declare that they have no competing interests.

## Authors’ contributions

FM is the PI of the EPICAP study. She conceived the study, designed the protocol and is in charge of the overall coordination of the study. She wrote the manuscript. AA has been the study monitor for the first 18 months and HR is the current study monitor of the EPICAP study. CM and PLP are in charge of the biological samples (EDTA tubes for cases and controls). FI, BL and RT are the referent urologist in the different health care department. They federated all urologists who work with them in their department. BL (urologist) is in charge of medical/histological validation of the cases in the different health care department. PJL is in charge of the biological samples (dry tubes for cases and controls) and is responsible for the “prognostic part” of the study. XR (urologist) and BT (head of the Hérault Cancer Registry) are part of the steering committee which meets every 3-4 months. All authors read and approved the final manuscript.

## Pre-publication history

The pre-publication history for this paper can be accessed here:

http://www.biomedcentral.com/1471-2407/14/106/prepub

## Supplementary Material

Additional file 1 English summarized version of the EPICAP questionnaire. Click here for file

## EPICAP Study Group

Urologists Didier Ayuso, Bruno Segui (Centre Hospitalier Bassin de Thau, Sète, France), Alain Guillaume, Jean-Paul Constans, Olivier Delbos, Pierre Lanfray, Damien Rizet, Etienne Cuénant (Cabinet Urologie du Polygone, Montpellier, France), Michel Locci (Centre Hospitalier, Béziers, France), Etienne Cuénant (Clinique Ste Thérèse, Sète, France), Nicolas Drianno, Bernard Marc, Paulo Soares (Polyclinique Saint Privat, Béziers, France), Antoine Faix, Samer Abdel Hamid, Bruno Segui (Service urologie, Clinique Beau Soleil, Montpellier, France), Samer Abdel Hamid(Clinique Saint Louis, Ganges, France), Laurent Cabaniols, Maxime Robert, Thibaut Murez, Grégoire Poinas, Nicolas Korahanis (Service d’Urologie, Hôpital Lapeyronie, Centre Hospitalo-Universitaire de Montpellier, France), Christian Prad, Nadine Soller (Registre des tumeurs de l’Hérault, Montpellier, France).

Pathologists : Marc Bernard, Didier Brel, Lysiane Schweizer, Philippe Nayraud, Claire Lecam-Savin (Béziers), Roland Daniel, Jean Baptiste Perdigou, Chantal Compan, Mireille Granier, Agnès Granier, Jean Louis Bouzigues, Elisabeth Broquerie, Joëlle Simony, Frédéric Bibeau, Pierre Baldet, Isabelle Serre (Montpellier), Majida Esslimani-Sahla, Marie Laure Gaume (Sète).

Biologists: Frédéric Montels (Service de Biologie Médicale, Institut du Cancer de Montpellier, Montpellier, France), Mr Dumas (Laboratoire de biologie, Béziers, France), Mrs Buono (Laboratoire de biologie, Sète, France), Mrs Bonnefille (Laboratoire de biologie, Lodeve, France), Mr Ruiz(Laboratoire de biologie, Lunel, France), Mr Paleirac (Laboratoire de biologie, Clermont-l’Hérault, France). All authors have read the manuscript, made contributions and approved the final text.
