# Supplementary material for: Epidemiological study of prostate cancer (EPICAP): a population-based case–control study in France
Source: BMC Cancer. 2014 Feb 19;14:106. doi: 10.1186/1471-2407-14-106 (PMC3936778; doi:10.1186/1471-2407-14-106)
Supplement: Additional file 1 — English summarized version of the EPICAP questionnaire. [file 1471-2407-14-106-S1.docx]

**English summarized version of the EPICAP questionnaire**

Section A: Socio-economic characteristics

Section B: Perinatal factors

Section C: Puberty, hormonal maturation, hormonal treatments

Section D: Fertlity

Section E: Lifetime anthropometry

Section F: Metabolic factors

Section G: Allergy/Atopy

Section H: Personal history of infectious and inflammatory diseases

Section I: Personal history Sexually Transmitted Infections

Section J: Personal history Viral infections

Section K: Personal history of vaccinations

Section L: Personal history of chronic diseases (inflammatory and autoimmune)

Section M: Medications

Section N: History of cancer screening

Section O: Family history of cancer in first- and second-degree relatives

Section P: Lifetime alcohol and tobacco consumption

Section Q: Lifetime physical activity

Section R: Chronotype scale (Horne and Ostberg)

Section S: Lifetime sleep duration

Section T: Residential history

Section U: Occupational history

Section V: Food frequency questionnaire
